# Supplementary material for: Nuclear Nestin deficiency drives tumor senescence via lamin A/C-dependent nuclear deformation
Source: Nat Commun. 2018 Sep 6;9:3613. doi: 10.1038/s41467-018-05808-y (PMC6127343; doi:10.1038/s41467-018-05808-y)
Supplement: Supplementary file 4 — Supplementary Information [file 41467_2018_5808_MOESM4_ESM.docx]

**Supplementary Information**

**Nuclear Nestin deficiency drives tumor senescence via lamin A/C-dependent nuclear deformation**

**Zhang et al.**

**
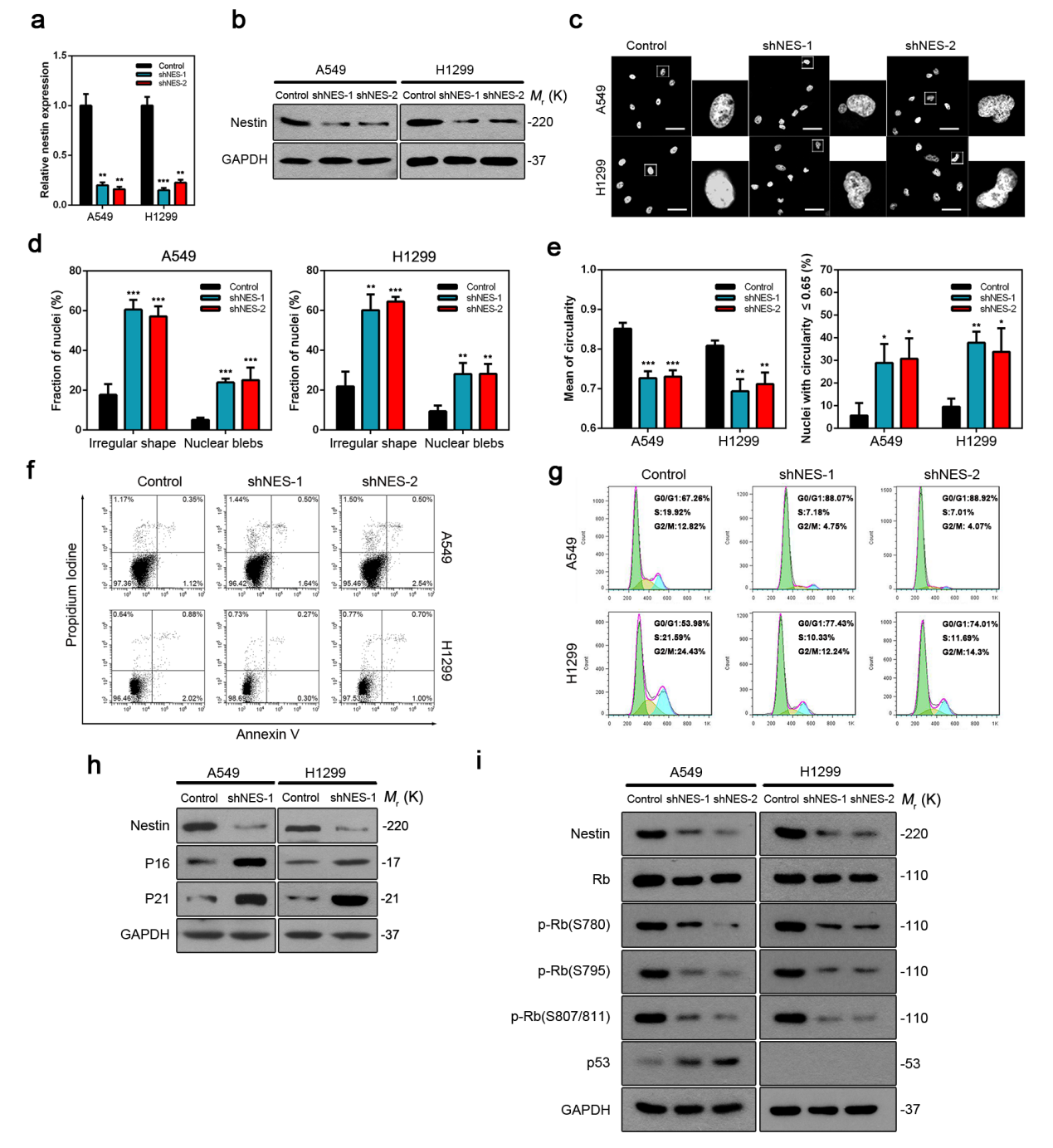
Supplementary Figure 1. Nestin knockdown induces cellular senescence.** (**a**) qPCR anaysis of Nestin expression in cells transfected with the indicated shRNAs. (**b**) Immunoblotting analysis of Nestin-depleted cells compared with negative controls. (**c**) The nuclear shapes of control and Nestin-knockdown cells observed with DAPI staining. Scale bars, 50 μm. (**d**) Analysis of abnormally shaped nuclei and nuclear blebs. (**e**) Nuclear circularity was analyzed using the Cellprofiler software. The panels showed mean circularities (left) and percentage of cells with deformed nuclei (circularity ≤ 0.65) from three independent experiments (right). (**f**) Flow cytometry analysis of cell apoptosis, with the Annexin V+PI+ and Annexin V+PI− populations taken as representing apoptotic cells. (**g**) Flow cytometry analysis of cell cycle arrest in lung cancer cell lines. (**h**) Immunoblotting anaysis of p16 and p21, in Nestin-knockdown and control cells. (**i**) Immunoblotting anaysis of p53 and pRb, in Nestin-knockdown and control cells. The quantified results were presented as mean ± SEM of three independent experiments, as assessed using unpaired t-test (**a,d,e**). *P <0.05, **P <0.01, ***P <0.001.

**
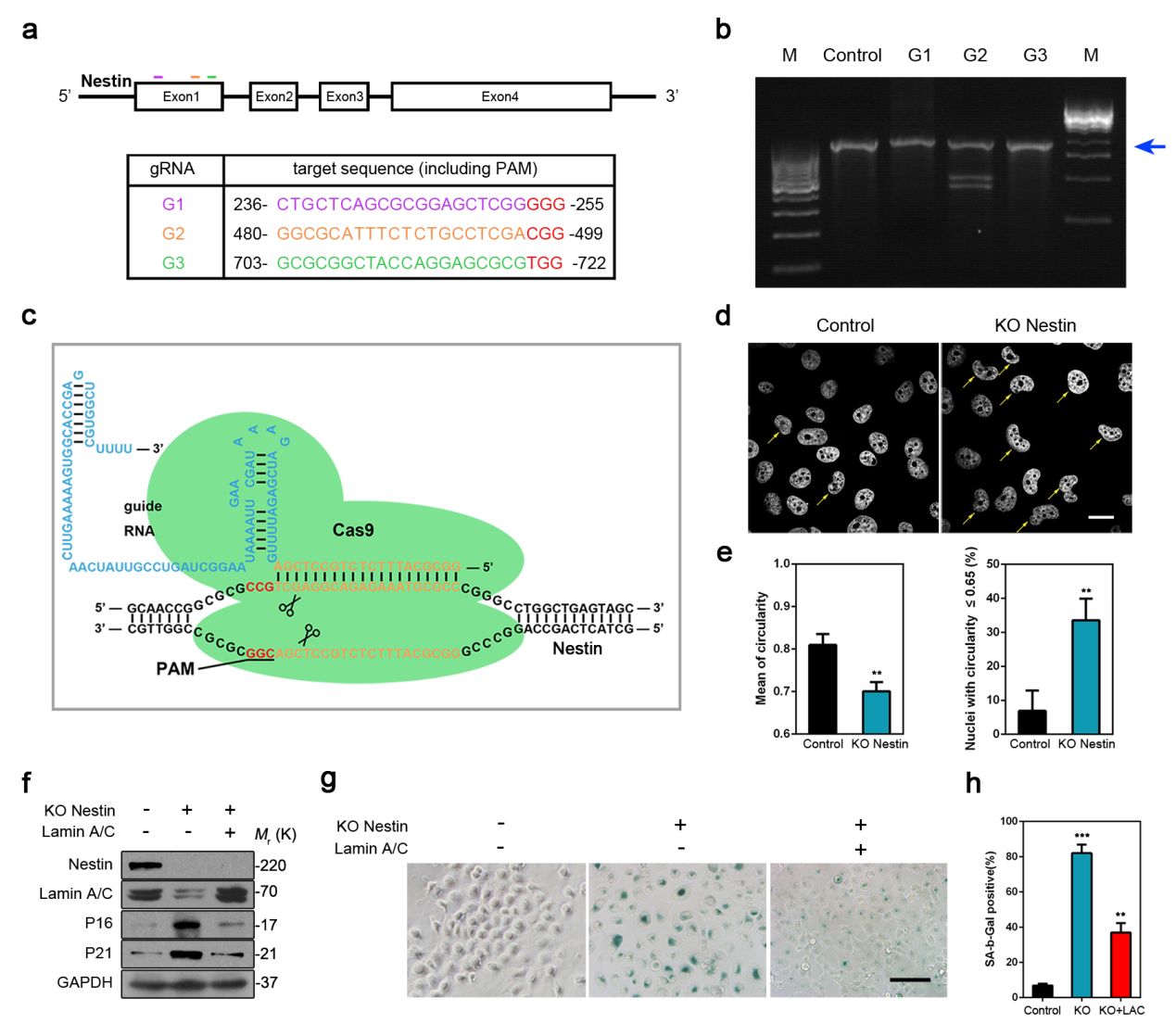
**

**Supplementary Figure 2. Effects of Nestin knockout on nuclear shape, lamin A/C stability, and cellular senescence in cancer cell lines.** (**a**) Three sgRNAs were designed to target 20 bp of Nestin exon 1. The sequences of each gRNA (G1, G2, and G3) were shown with the PAM (NGG) sequences highlighted in red. (**b**) HEK293T cells were transfected with vectors co-expressing sgRNA and Cas9, and mutation frequencies were measured using the T7E1 assay after 60 hr. Blue arrow indicated the expected size for the uncut (no mismatch) PCR product. (**c**) Schematic structure of the Nestin gene-targeting CRISPR-Cas9 system. The most efficient target sequence selected from three sgRNAs was shown in orange. (**d,e**) The nuclear shapes of control and Nestin-knockout (KO Nestin) H1299 cells were observed with DAPI staining (**d**) and analyzed with the Cellprofiler software (**e**). Yellow arrows showed alterations in nuclear morphology. Scale bars, 20 μm. (**f**) The protein levels of Nestin, lamin A/C, p16, and p21 were evaluated by immunoblotting analysis in control and Nestin-knockout cells. (**g**) SA-β-gal staining was used to identify the frequency of senescent cells in vitro (left panel, ×400). Scale bars, 100 μm. (**h**) Quantification of the SA-β-gal-positive cells in **g**. For all quantifications, the data represented the mean ± SEM of three independent experiments, as assessed using unpaired t-test. *P <0.05, **P <0.01, and ***P <0.001.

**
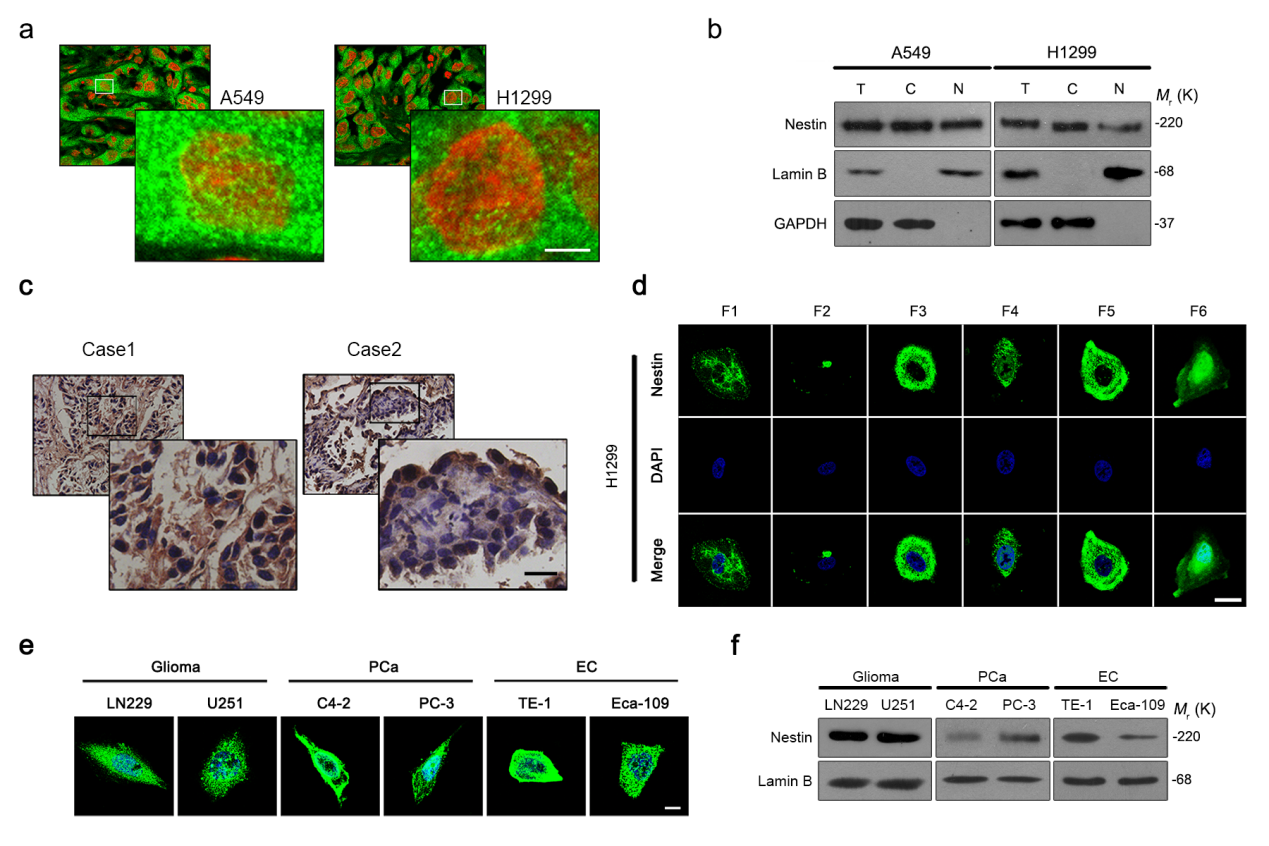
**

**Supplementary Figure 3. Nestin localizes to the cell nuclei of adenocarcinoma tissues and various cancer cells.** (**a**) Subcutaneous tumors derived from A549 or H1299 cells were immunostained with anti-Nestin (green) antibody and DAPI (red). Scale bars, 10 μm (n=3). (**b**) Immunoblotting analysis of Nestin expression in subcutaneous tumors (T, total cell lysates; C, cytoplasmic cell lysates; N, nuclear cell lysates). (**c**) Immunohistochemical staining of Nestin in lung cancer clinical samples. Scale bars, 30 μm (n=2). (**d**) Vectors encoding various versions of GFP-Nestin were transfected into H1299 cells, and the cells were observed by fluorescent microscopy. Nuclei were marked with DAPI (blue). Scale bars, 20 μm. (**e**) Confocal images of Nestin immunostaining (green) in several cancer cell lines (PCa, prostatic cancer; EC, esophageal cancer). Scale bars, 10 μm. (**f**) Immunoblotting for Nestin in nuclear fractions obtained from several cancer cell lines.


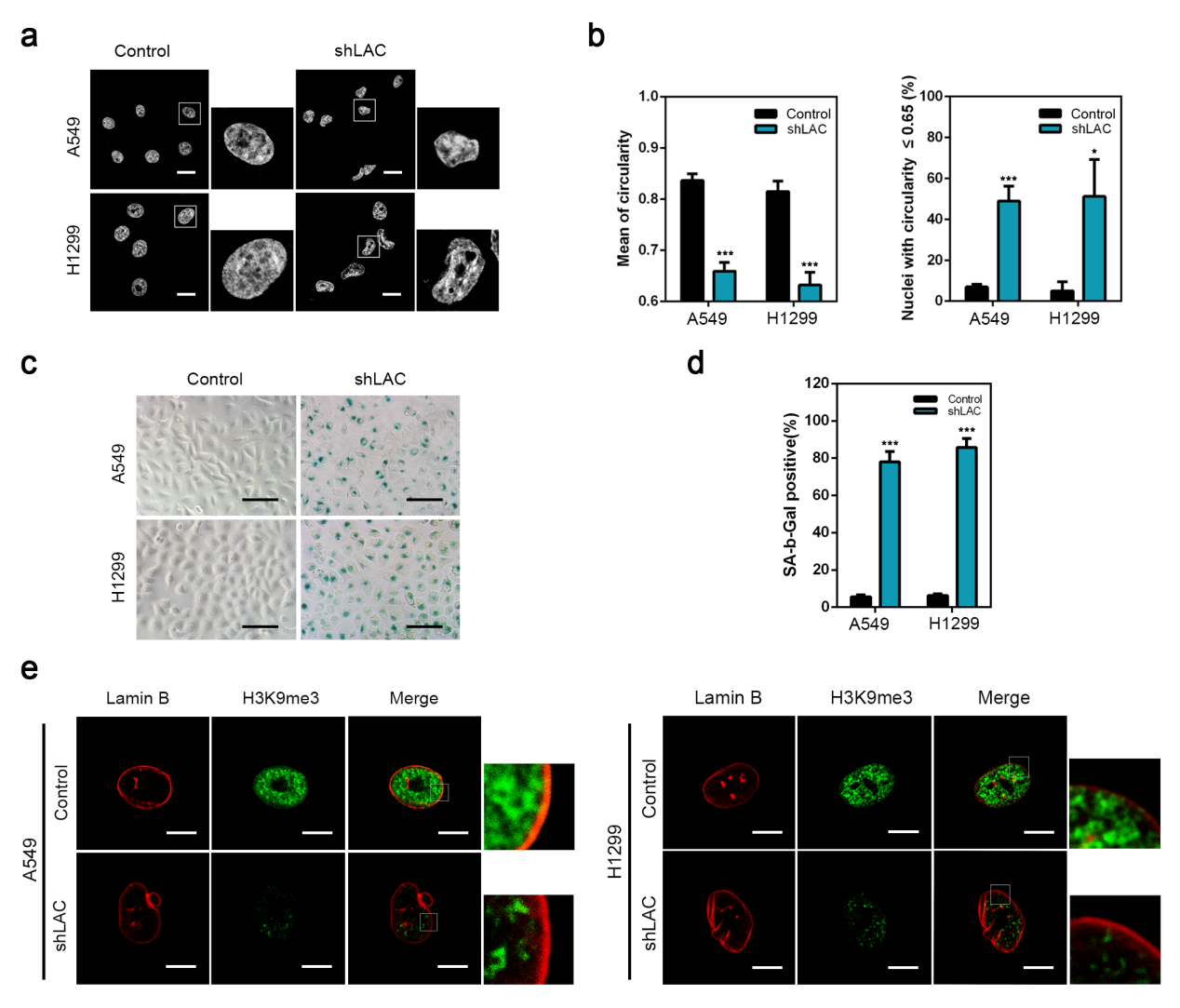


**Supplementary Figure 4. Lamin A/C depletion drives cancer cell senescence.** (**a**) The nuclear shapes of control and lamin A/C-knockdown cells, as observed with DAPI staining. (**b**) Nuclear circularity was analyzed with the Cellprofiler software, which quantified the mean circularity and percentage of cells with deformed nuclei in lamin A/C-knockdown and control cells. (**c**) SA-β-gal staining was used to identify the frequency of senescent cells in vitro (left panel, ×400). Scale bars, 100 μm. (**d**) Quantification of the SA-β-gal-positive cells in **c**. (**e**) Immunofluorescence staining of H3K9me3 (green) and lamin B (red) in control and lamin A/C-knockdown cells. Scale bars, 10 μm. The quantified results were presented as mean ± SEM of three independent experiments, as assessed using unpaired t-test (**b**,**d**). *P <0.05, **P <0.01, ***P <0.001.

**
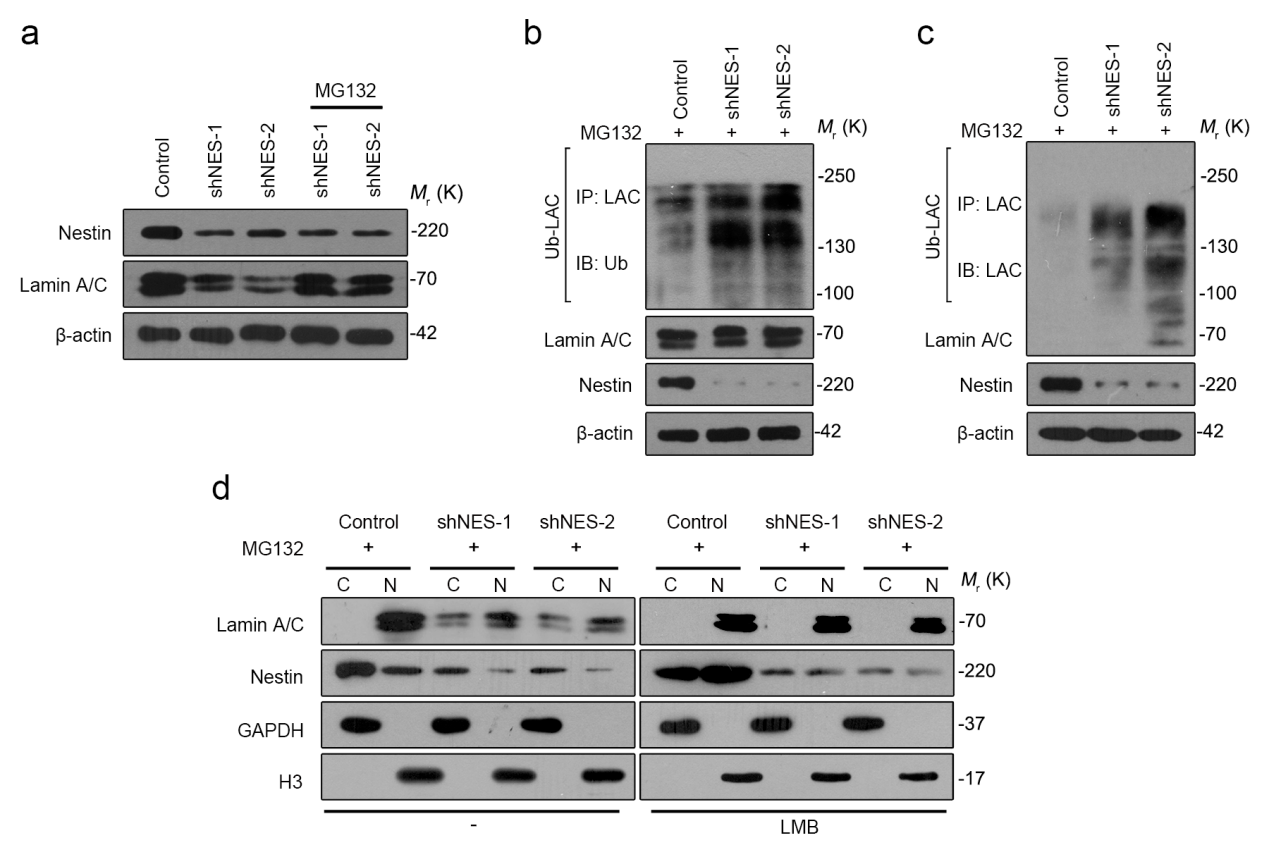
**

**Supplementary Figure 5. Nestin protects lamin A/C from proteasomal degradation.** (**a**) Control and Nestin-knockdown H1299 cells were treated with or without MG132 (20 μM) for 6 hr, and proteins were extracted and subjected to immunoblotting. (**b,c**) The effects of Nestin knockdown on ubiquitination of lamin A/C were analyzed by in vivo ubiquitination assays. H1299 cells of control or Nestin-knockdown were treated with MG132 (20 μM, 6 hr) before harvest. Lamin A/C was immunoprecipitated with anti-lamin A/C antibody and immunoblotted with anti-Ub antibody (**b**) or anti-lamin A/C antibody (**c**). (**d**) Immunoblotting analysis of lamin A/C distribution in nucleus and cytoplasm. H1299 cells transfected with the indicated constructs were treated with MG132 (20 μM, 2 hr) and then treated with or without 25 ng/ml LMB. Four hours later, the cells were harvested and fractionated, and the cellular fractions were immunoblotted with the indicated antibodies (C, cytoplasmic cell lysates; N, nuclear cell lysates).


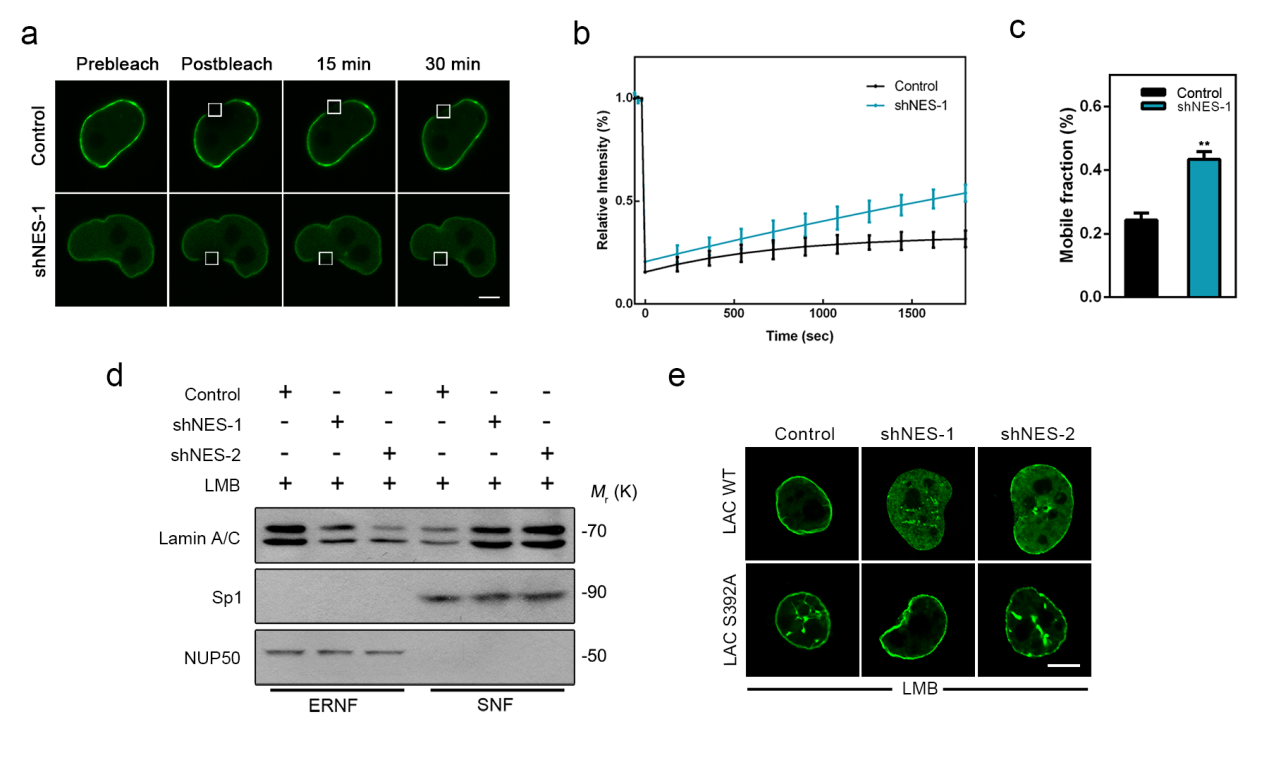


**Supplementary Figure 6. Phosphorylation by Cdk5 drives the degradation of lamin A/C after Nestin knockdown.** (**a,b**) FRAP anaysis of GFP-lamin A/C in control and Nestin-knockdown H1299 cells. (**a**) A typical FRAP profile. Scale bars, 5 μm. (**b**) The mean normalized FRAP data obtained from bleached regions (n=5). (**c**) The average mobile fractions of lamin A/C proteins were calculated from the FRAP data in **b** (n=5). (**d**) Immunoblotting analysis of lamin A/C distribution in SNF and ERNF. All groups of H1299 cells were treated with 25 ng/ml LMB for 4 hr. Anti-Sp1 and anti-NUP50 antibody confirmed equal protein loading and the absence of cross-contamination. (**e**) Confocal microscopy was employed to determine the subcellular localization of WT GFP-lamin A/C, compared with GFP-lamin A/C-S392A. H1299 cells transfected with the indicated constructs were treated with 25 ng/ml leptomycin B (LMB) for 6 hr, and then harvested, fixed, and stained with DAPI (blue). Scale bars, 10 μm. The quantified results were presented as mean ± SEM of five independent experiments (**b,c**), as assessed using unpaired t-test (**c**). *P <0.05, **P <0.01, ***P <0.001.


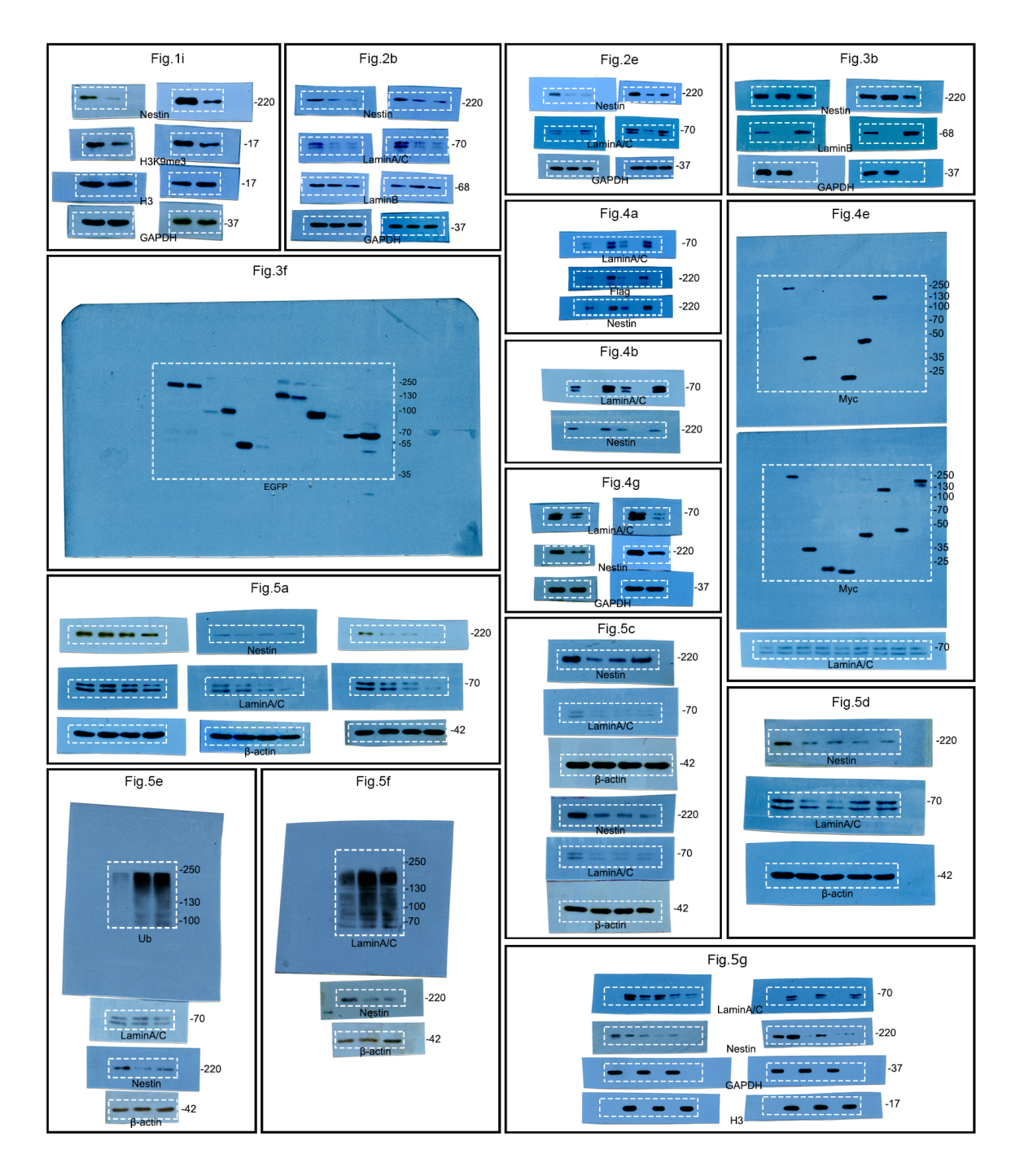


**Supplementary Figure 7. Full length images of immunoblots.**

**
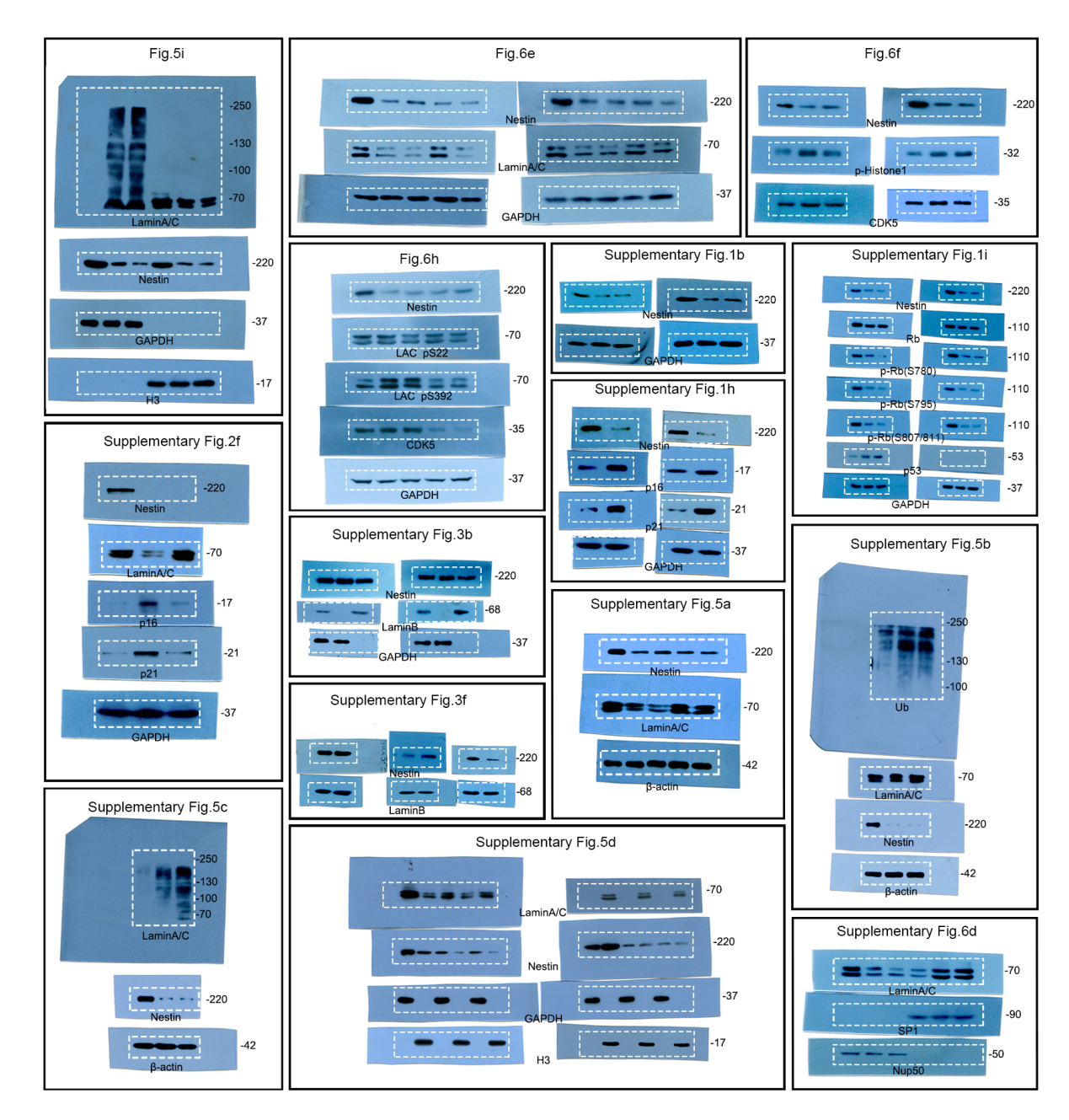
**

**Supplementary Figure 7. Continued.**

| **Supplementary Table 1 Target sequences of shRNAs. Related to Experimental Procedures** | |
| --- | --- |
| **Name** | **Sequence (5′ to 3′)** |
| Nestin shRNA#1 | **GGCAGACATCATTGGTGTTAA** |
| Nestin shRNA#2 | **GGCTAGTCCCTGCCTGAATAA** |
| Lamin A/C shRNA | **CTCATCTATCTCAATCCTAAT** |
| Cdk5 shRNA#1 | **CCTGAGATTGTAAAGTCATTT** |
| Cdk5 shRNA#2 | **TTTATGAAATTAAATAAAGTCCA** |

| **Supplementary Table 2 Primer used to amplify the human transcripts or genome DNA during PCR. Related to Experimental Procedures** | | |
| --- | --- | --- |
| **Gene (human)** | **Sequence (5′ to 3′)** | **Application** |
| *LMNA* | **Upper: AGCAAAGTGCGTGAGGAGTT**  **Lower: TCAGGTCACCCTCCTTCTTG** | **qPCR** |
| *LMNB1* | **Upper: AAGCAGCTGGAGTGGTTGTT**  **Lower: TTGGATGCTCTTGGGGTTC** | **qPCR** |
| *IL6* | **Upper: GGTACATCCTCGACGGCATCT**  **Lower: GTGCCTCTTTGCTGCTTTCAC** | **qPCR** |
| *CXCL8* | **Upper: CTGGCCGTGGCTCTCTTG**  **Lower: CCTTGGCAAAACTGCACCTT** | **qPCR** |
| *NES* | **Upper: CTGCTACCCTTGAGACACCTG**  **Lower: GGGCTCTGATCTCTGCATCTAC** | **qPCR** |
| *GAPDH* | **Upper: GTCGGAGTCAACGGATTT**  **Lower: GGAATCATATTGGAACATGTAAACC** | **qPCR** |
| *NES* | **Upper: TACTCCCACCCCGCCCCGCCCCGTCATTG**  **Lower: GCAGCCGCTCCTGCCAGCGGCCCTCCAACC** | **PCR** |

**Supplementary Table 3 Primary and secondary antibodies**

| **Product** | **Catalogue Number** | **Supplier** | **dilution** |
| --- | --- | --- | --- |
| **Primary antibodies:** |  |  |  |
| **WB:** |  |  |  |
| **rabbit anti-Cdk5** | **sc-173** | **Santa Cruz** | **1:500** |
| **mouse anti-Nestin** | **611658** | **BD Biosciences** | **1:1000** |
| **mouse anti-β-actin** | **3700s** | **Cell Signaling Technology** | **1:1000** |
| **goat anti-lamin B** | **sc-6216** | **Santa Cruz** | **1:500** |
| **mouse anti-Ub** | **sc-8017** | **Santa Cruz** | **1:500** |
| **rabbit anti-lamin A/C pS22** | **2026S** | **Cell Signaling Technology** | **1:1000** |
| **rabbit anti-lamin A/C pS392** | **ab58528** | **Abcam** | **1:1000** |
| **rabbit anti-pH1** | **ab4270** | **Abcam** | **1:1000** |
| **rabbit anti-GAPDH** | **2118s** | **Cell Signaling Technology** | **1:5000** |
| **rabbit anti-P21** | **2947s** | **Cell Signaling Technology** | **1:1000** |
| **rabbit anti-Sp1** | **sc-59** | **Santa Cruz** | **1:500** |
| **rabbit anti-NUP50** | **ab151567** | **Abcam** | **1:1000** |
| **mouse anti-lamin A/C** | **ab8984** | **Abcam** | **1:1000** |
| **rabbit anti-H3** | **ab1791** | **Abcam** | **1:2000** |
| **rabbit anti-H3K9me3** | **ab8898** | **Abcam** | **1:1000** |
| **rabbit anti-P16INK4a** | **ab108349** | **Abcam** | **1:1000** |
| **mouse anti-DYKDDDDK** | **8146** | **Cell Signaling Technology** | **1:1000** |
| **mouse anti-cMyc** | **2276s** | **Cell Signaling Technology** | **1:1000** |
| **IP:** |  |  |  |
| **mouse anti-DYKDDDDK** | **8146** | **Cell Signaling Technology** | **1:100** |
| **goat anti-lamin A/C** | **sc-6215** | **Santa Cruz** | **1:50** |
| **ICC:** |  |  |  |
| **rabbit anti-Nestin** | **ab27952** | **Abcam** | **1:200** |
| **rabbit anti-Nestin** | **ABD69** | **Millipore** | **1:200** |
| **rabbit anti-H3K9me3** | **ab8898** | **Abcam** | **1:200** |
| **goat anti-lamin B** | **sc-6216** | **Santa Cruz** | **1:100** |
| **mouse anti-lamin A/C** | **ab8984** | **Abcam** | **1:200** |
| **rabbit anti-lamin A/C pS392** | **ab58528** | **Abcam** | **1:200** |
| **rabbit anti-lamin A/C pS22** | **2026S** | **Cell Signaling Technology** | **1:200** |
| **IHC:** |  |  |  |
| **rabbit anti-Nestin** | **ab27952** | **Abcam** | **1:200** |
| **rabbit anti-Nestin** | **ABD69** | **Millipore** | **1:200** |
| **Secondary antibodies:** |  |  |  |
| **WB:** |  |  |  |
| **anti-mouse IgG HRP-linked Ab** | **7076** | **Cell Signaling Technology** | **1:5000** |
| **anti-rabbit IgG HRP-linked Ab** | **7074** | **Cell Signaling Technology** | **1:5000** |
| **anti-goat IgG HRP-linked Ab** | **Ab6885** | **Abcam** | **1:3000** |
| **ICC:** |  |  |  |
| **goat anti-mouse IgG Alexa 488** | **A11001** | **Invitrogen** | **1:500** |
| **goat anti-rabbit IgG Alexa 488** | **A11008** | **Invitrogen** | **1:500** |
| **goat anti-rabbit IgG Alexa 555** | **A21428** | **Invitrogen** | **1:500** |
| **goat anti-mouse IgG Alexa 555** | **A21422** | **Invitrogen** | **1:500** |
| **donkey anti-goat IgG Alexa 594** | **A11058** | **Invitrogen** | **1:500** |
