# Supplementary material for: Nuclear Nestin deficiency drives tumor senescence via lamin A/C-dependent nuclear deformation
Source: Nat Commun. 2018 Sep 6;9:3613. doi: 10.1038/s41467-018-05808-y (PMC6127343; doi:10.1038/s41467-018-05808-y)
Supplement: Supplementary file 5 — Description of Additional Supplementary Files [file 41467_2018_5808_MOESM5_ESM.pdf]

Description of Additional Supplementary Files

File Name: Supplementary Movie 1
Description: Super-resolution 3-D image of the subcellular localization of Nestin and lamin A/C in A549

File Name: Supplementary Movie 2
Description: Super-resolution 3-D image of the subcellular localization of Nestin and lamin A/C in H1299
